# Why canines induce resorption of neighboring roots? An imaging correlation

**DOI:** 10.1590/2177-6709.24.1.027-033.oin

**Published:** 2019

**Authors:** Alberto Consolaro, Omar Hadaya, Taisa Maeshiro Estorce

**Affiliations:** 1 Universidade de São Paulo, Faculdade de Odontologia de Ribeirão Preto, Programa de Pós-Graduação de Odontopediatria (Ribeirão Preto/SP, Brazil).; 2 Universidade de São Paulo, Faculdade de Odontologia de Bauru (Bauru/SP, Brazil).; 3 Private practice (Maringá/PR, Brazil).; 4 Universidade de Guarulhos, Curso de Especialização em Ortodontia (Guarulhos/SP, Brazil).

**Keywords:** Root resorption, Dental follicle, Unerupted teeth, Impacted canines

## Abstract

Despite the explanations about the mechanisms and reasons why dental follicles of unerupted maxillary canines do not cause root resorption in neighboring teeth, questions remain about the time expected for this event and the lack of protocols for preventive clinical management, which may serve as insights for further studies. Here, these mechanisms are correlated with imaging findings of CT scans and 3D reconstructions of a typical clinical case.

## RESUMO

Apesar das explicações dos mecanismos e dos porquês os folículos pericoronários dos caninos superiores não irrompidos podem reabsorver as raízes dos dentes vizinhos, questiona-se a inexistência de previsibilidade do tempo em que isso ocorre e a ausência de protocolos de condutas clínicas preventivas, como *insights* para novas pesquisas. Correlaciona-se, também, esses mecanismos com os aspectos imaginológicos de cortes tomográficos e reconstruções 3D de um caso clínico característico.

## INTRODUCTION

The most frequent cause why a maxillary canine does not erupt is lack of space between maxillary lateral incisor and first premolar, so that the canine can align its long axis to that of these other teeth and emerge in the dental arch. 

Despite this lack of space, the resulting forces generated by bone growth in the anterior region of the maxilla and premaxilla, also called vectors, may block normal canine eruption path, and the canine may:^1^

**Figure 1:**
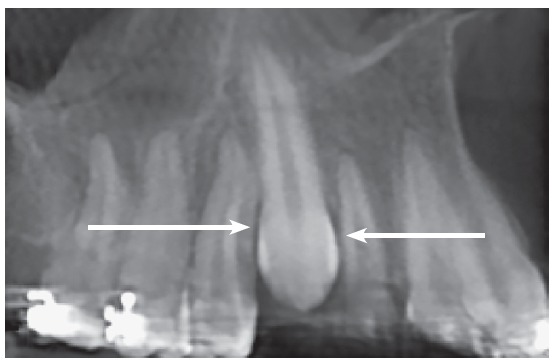
Radiographic reconstruction showing region of unerupted canine between maxillary lateral incisor and first premolar, in special position of canine crown, close to canine roots. Radiolucent (hypodense) area around canine crown extending from periodontal space of neighboring roots (arrows). Space measures same as canine mesiodistal width, but is not large enough for dental follicle.

**Figure 2:**
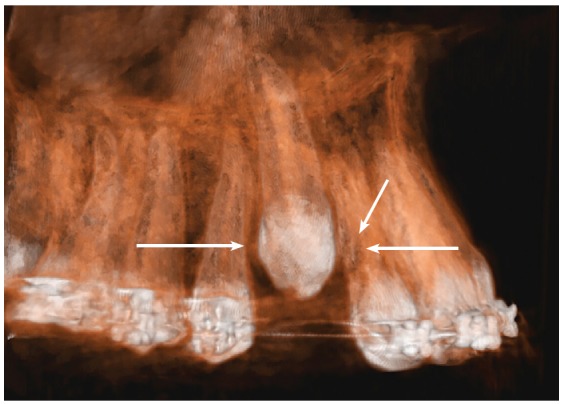
Buccal view of 3D reconstruction of maxillary canine region in same patient as in Figure 1. Hypodense pericoronal space extends from periodontal space of neighboring roots, and root surfaces of both teeth are irregular (arrows). Space measures same as canine mesiodistal width, but is not large enough for dental follicle.

**Figure 3:**
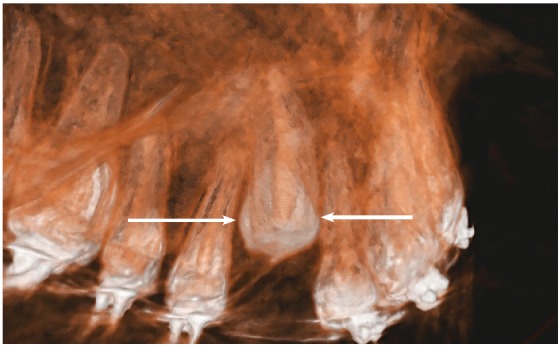
Palatal view of 3D reconstruction of maxillary canine region in same patient as in Figure 2. Hypodense pericoronal space extends from periodontal space of neighboring roots, and root surfaces of both teeth are irregular (arrows).

**Figure 4:**
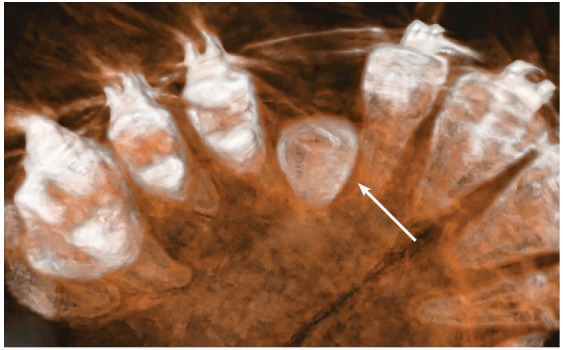
Occlusal view of 3D reconstruction of maxillary canine region in same patient as in Figure 3. Hypodense pericoronal space is clearly restricted in this region (arrow).

1) Erupt ectopically on the buccal surface or on the palate.
2) Not erupt and remain intraosseously lodged in a variety of possible positions, with no direct relation to the other teeth.
3) Erupt partially in the oral cavity, between the maxillary lateral incisor and the first premolar, still in the dental arch, but in infraocclusion.
4) Remain for a long time between the maxillary lateral incisor and the premolar, as in Figures 1 to 4.

If the canine remains between the roots of the lateral incisors and upper premolars for long periods of time, one may ask:

## 1. Why does the canine not erupt, since there is enough space for its crown to fit in the dental arch?

An analogy should be used here: the engine of a car is internal, placed inside the body of the car; in contrast, the dental follicle, the “engine” of a tooth that produces the eruption forces and movements, is external to the structure of the crown. For the car to move, the engine has to move too, and it does so easily, because it is inside the car; however, for the tooth to move, it needs space for its engine too, that is, for its dental follicle, which is external to the crown. There should be space for both, the canine crown and the canine dental follicle; if there is space only for the crown, the tooth will be “stuck”, or locally impacted, as in Figures 1 and 2.

Tooth eruption does not depend on the root and its stage of formation, but on its dental follicle^2^. Studies showed that the experimental removal of the root does not affect eruption, but the removal of the dental follicle, in contrast, blocks it.^1^ ^,^ ^2^ The dental follicle, or dental sack, is the fibrous connective tissue sack that attaches to the crown firmly by the reduced epithelium, which formed enamel. Connective tissue still has many epithelial cords and islands of the dental lamina. The cells of the dental follicle, particularly epithelial cells, release several chemical mediators to the neighboring bone cells. Of these mediators, the epithelial growth factor (EGF) stimulates pericoronal bone resorption and opens the space for the tooth to erupt into the oral cavity.^3^ ^-^ ^6^

If the space reserved for the canine in the dental arch is not about 1.5 times canine mesiodistal width, there will be no space to fit the dental follicle, and the canine will not move into its place, not even when traction forces are used. Some authors and dentists consider that this space of 1.5 times the mesiodistal width is too large. In fact, this has been determined because the dental follicle of the maxillary canine, because of this tooth specific anatomy, is more bulging and wider than the other teeth in the neighboring regions. However, eruption may be successful even in a smaller space, depending on the clinical conditions of each specific case. 

The force naturally generated by the growth vectors, or applied orthodontically, stimulates cells to release mediators of bone resorption. The follicle is necessary for this, as it moves at the same time as the canine crown. Regardless of the characteristics of forces, the follicle has to move together with the tooth for eruption to occur. Osteoclasts are cells that open the path for eruption by resorbing the neighboring bone; they are part of this environment and work only in the presence of the connective tissue found in the dental follicle.

In most clinical cases, it is not necessary to open the space between the lateral incisor and the premolar or to move the tooth orthodontically when the space is 1.5 times the mesiodistal width of the canine, in which case the canine erupts spontaneously and naturally. It is important to wait a few weeks before using a bracket for traction.

In some clinical cases, maybe only a few, the canine may take too long to erupt or not erupt at all, probably because of an inadequate position, as it may be at a very acute angle and/or horizontal position, which then requires the use of traction to guide it to the path to be followed. Studies should be conducted to determine a waiting time protocol for this situation: how much time should we wait (days, weeks, months) after opening the space in the dental arch.

### Precautions in surgery for canine traction

During surgery to place the bracket, some precautions should be taken:

1) As the enamel has to be exposed as much as necessary for the procedure, avoid manipulating the region of the cementoenamel junction, which is very delicate and has macroexpositions of dentin. Such manipulation may induce bone resorption in the cervical region in the future, after the tooth has already erupted into the occlusal plane; and this is not desired, although external cervical resorption may occur sometimes.^7^ ^-^ ^10^
2) Do not apply to much acid to condition enamel before applying the resin, because it may act in the same way as the manipulation at the cementoenamel junction, which is very delicate and where dentin is exposed, which may lead to the same type of future external cervical resorption aforementioned.^6^ ^-^ ^10^
3) Check whether the canine is not stuck because of its proximity to the roots of neighboring teeth, which requires specific mechanical actions to move it from this initial position and, after that, to take it to the ideal location.
4) At this moment, do not use surgically assisted luxation, which should be used only in the third phase of canine traction. This procedure should only be used in cases with a final clinical and imaging diagnosis of canine ankylosis.^6^ ^-^ ^10^

If the canine does not erupt even if the space is 1.5 times the mesiodistal width of the tooth, enough for eruption, and when traction cannot pull it out, we should review specific canine radiographs and CT scans to check whether there is ankylosis. This is a considerable visual exercise, as ankylosis is only seen when it affects at least 20% of the root surface. 

In several cases, the diagnosis will be only clinical, and not based on imaging studies. If the canine has enough space to erupt and traction was not successful, an early diagnosis of ankylosis may be made, even when it is not seen on imaging studies. At this point, the last maneuver to bring the canine out is performed, without displacing it from the alveolus, because such displacement would characterize an autogenous transplant or reimplantation.^1^ ^,^ ^6^ ^-^ ^10^

An important note here: in case of unerupted canines, always think three-dimensionally, because this helps to determine why its eruption is delayed or blocked. It also supports the choice of an adequate mechanical maneuver to move it out of its initial position and take it into the desired location.

## 2. When and why root resorption affects teeth neighboring the unerupted maxillary canine?

The time that a maxillary canine takes to begin external inflammatory resorption on the external surface of the roots of neighboring teeth is a clear gap in our understanding of it. How long after normal time for maxillary canine eruption does resorption of neighboring teeth occur? Which factors significantly affect length of time to the beginning of neighboring root resorption? Such predictability may exist, but it has not been methodologically determined yet.

**Figure 5:**
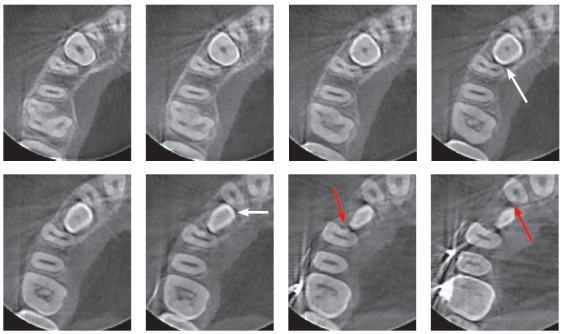
Axial CT scans of cervical third of impacted maxillary canine of same patient as in Figures 1 to 4. Hypodense pericoronal space extends from periodontal spaces of neighboring tooth roots (white arrows) with slightly irregular surfaces, indicative of incipient external root resorption (red arrows). Space measures same as canine mesiodistal width, but is not large enough for dental follicle, and death of cementoblastic cells exposes region to osteoclasts.

Faced with clinical cases as the one shown in Figures 1 to 5, it is common to say that the canine is pushing neighboring teeth, or that the canine is touching the lateral incisor root. When that is not the case, some may say that the canine crown is resorbing the neighboring root.

Teeth do not touch each other.^11^ Before this may happen, the canine dental follicle moves closer to the neighboring periodontal ligament, compresses vessels and promotes anoxia of cementoblasts, which then necrotize (Figs 2 and 5).

In this region, there will be several root areas exposed to mineralized tissues, such as the cementum and the dentin, which attract osteoclasts that are deposited on the root, as actual “sucking-cups”. They then start root resorption with the support of osteoblasts and macrophages found in the dental follicle. 

These cells and root resorption require a blood supply to carry oxygen and glucose, so that they can function. There will always be some vascularized connective tissue between a canine and a site of neighboring root resorption. The mineralized tissue of the crown or the enamel will never be directly on the mineralized tissue of the root undergoing resorption; there will always be some connective tissue between them, a tissue rich in osteoclasts and other cells.^11^

When they “see” root areas of exposed mineralized tissue on the root, the osteoclasts migrate, attach to the surface and are stimulated to resorb it by bone reabsorption mediators found in follicular tissue.

In the dental follicle, there are several reabsorption mediators, particularly EGF, which is responsible for the activation of other mediators.^12^ EGF, as its name implies, originates in any epithelia. In the case of the dental follicles, there is the reduced enamel epithelium, which covers all the normal enamel and the epithelial islands of residual dental lamina, which was fragmented soon after it originated teeth in the deeper planes of the jaws.

Bone resorption mediators found in follicular tissues are there to promote eruption and open the path for the tooth to reach the occlusal plane. The dental follicle, and not the tooth root, is the organ responsible for tooth eruption, contrary to what was believed for many years.^2^

The images shown here should be understood based on these considerations and explanations. The CT scans, as well as the 3D reconstructions, reveal the presence of a hypodense space between the unerupted canine crown, the bone and the neighboring roots (Figs 1 to 5). 

In several cases, resorption is not detectable on periapical radiographs, but CT scans reveal them.^13^ ^,^ ^14^ Conventional periapical and panoramic radiographs show that 12.5% of the cases lead to incisor resorption, but dental CT scans double the number of cases detected.^13^ ^,^ ^14^ Finally, although images may supposedly show contact between teeth, with or without associated root resorption, microscopic examination will reveal a small band or membrane of connective tissue that is rich in active osteoclasts.^11^ Teeth and mineralized tissue alone are not able to resorb another tooth.

## FINAL CONSIDERATIONS

The main functions of a dental follicle are:

1) Protect enamel from resorption by osteoclasts.
2) Prevent bone from forming directly on the surface of enamel.
3) Essentially, promote tooth eruption by releasing mediators.
4) Give rise to primary junctional epithelia, as it fuses with the oral mucosa and allows the tooth to erupt into the oral cavity without exposure to the highly contaminated external environment.

Maxillary canines, when unerupted, are epigenetically affected by several factors in its pathogenecity.^15^ These epigenetic factors, added to the already known biological potential and functions, suggest that the problems with maxillary canine eruption are predictable and that they may be prevented during craniomandibular growth. However, such predictability and prevention remain to be determined, especially in clinical studies to define management protocols.

In addition, when submitted to surgical procedures with or without bracket placement, epithelial and connective follicular tissues proliferate and regenerate immediately after that: the epithelium, in some hours, and the connective tissue, in some days. This biological characteristic also favors orthosurgical interception.

Because of all these properties, the dental follicle promotes resorption of neighboring roots when located very close to its periodontal ligament (Figs 1 to 5). The induction of bone resorption around it is part of its physiological nature, but it does not distinguish what is bone from what is tooth!
